# Clinical and Laboratory Studies of the Fate of Intranasal Allergen

**DOI:** 10.1371/journal.pone.0127477

**Published:** 2015-05-13

**Authors:** Janet Rimmer, Conceição Santos, Eija Yli-Panula, Virginia Noronha, Markku Viander

**Affiliations:** 1 Allergen Group, Woolcock Institute of Medical Research, Sydney, New South Wales, Australia; 2 Sydney Medical School, the University of Sydney, Sydney, New South Wales, Australia; 3 Department of Teacher Education, University of Turku, Turku, Finland; 4 Department of Medical Microbiology and Immunology, University of Turku, Turku, Finland; Cincinnati Children's Hospital Medical Center, University of Cincinnati College of Medicine, UNITED STATES

## Abstract

**Background:**

The precise way in which allergen is handled by the nose is unknown. The objective of this study was to determine recovery of Der p 1 allergen following nasal administration and to determine whether Der p 1 can be detected in nasal biopsies after natural exposure and nasal challenge to allergen.

**Methods:**

(1) 20 nonatopic non-rhinitics were challenged with Der p 1 and recovery was measured by ELISA in the nasal wash, nasal mucus and induced sputum up to 30 minutes. Particulate charcoal (<40 μm) served as control. (2) In 8 subjects (5 atopics), 30 to 60 minutes after challenge histological localisation of Der p 1 in the nasal mucosal epithelium, subepithelial mucous glands and lamina propria was performed. Co-localisation of Der p 1 with macrophages and IgE-positive cells was undertaken.

**Results:**

(1) Less than 25% of total allergen was retrievable after aqueous or particulate challenge, most from the nasal mucus during 1-5 min after the challenge. The median of carbon particles recovered was 9%. (2) Prechallenge Der p 1 staining was associated with the epithelium and subepithelial mucous glands. After challenge there was a trend for greater Der p 1 deposition in atopics, but both atopics and nonatopics showed increases in the number of Der p 1 stained cells and stained tissue compartments. In atopics, increased eosinophils, macrophages and IgE positive cells co-localized with Der p 1 staining.

**Conclusions:**

Der p 1 allergen is detected in nasal tissue independent of atopic status after natural exposure. After challenge the nose effectively retains allergen, which remains mucosally associated; in atopics there is greater Der p 1 deposition and inflammatory response than in nonatopics. These results support the hypothesis that nasal mucus and tissue act as a reservoir for the inhaled Der p 1 allergen leading to a persistent allergic inflammatory response in susceptible individuals.

## Introduction

It is increasingly recognised that host-environment interactions are important in the development and persistence of airway inflammation. The majority of allergenic and non-allergenic particles enter the respiratory tract via the nasal cavity but there is incomplete data on how the nose interacts with these particles. Most insoluble particles over 4 microns in diameter are caught on the superficial gel layer of the mucous blanket, and swept backwards to the nasopharynx by mucociliary clearance [1]. Soluble materials dissolve into the periciliary or sol phase of the mucous blanket. There is incomplete information on how intranasal allergen is handled, its mechanisms of penetration into the epithelium, its interaction with epithelial cells and the cells of the immune system, such as dendritic cells and subsequently the elimination of allergen.

The interaction between allergen and the epithelium may depend both on the physical characteristics of the allergen and also the active and passive properties of the epithelium [2, 3].

The barrier of the nasal respiratory mucosa comprises mucus formation by goblet cells and by submucosal glands, mucociliary clearance, the nasal epithelium with tight junctions and dendritic cells [4, 5, 6, 7, 8, 9]. The epithelial cells are no longer considered to act only as a physical barrier toward the inhaled allergen, but also to actively contribute to airway inflammation by detecting and responding to environmental factors [2, 10, 11, 12, 13, 14, 15]. The integrity of the epithelial barrier may be affected by atopic status [2, 3, 16, 17, 18, 19], mutations within the filaggrin gene [20] and a range of environmental factors such as pollution [9, 21] and infections [2, 22, 23].

Allergens have different strategies to pass across the epithelium. Proteolytic activity is a general feature of major allergens [2, 24]. Many dust mite, pollen, fungi and cockroach allergens are identified as cysteine or serine proteases able to degrade tight junction proteins, compromising epithelial integrity, thus facilitating allergen uptake by DCs in the subepithelial tissue [8, 17, 24]. In addition, direct uptake and transcytosis of Phl p 1 by airway epithelial cells in less than 2 hours has been shown suggesting that alternative mechanisms exist [25].

There are few studies on the fate of allergen exposed to the nasal cavity. Human studies using radiolabelled allergen (Par j 1) suggest that the majority of the allergen is cleared from the nasal cavity by the mucociliary pathway and is then swallowed. These studies also demonstrated that Par j 1 allergen inserted into the nose stays in the nasal mucosa of allergic subjects for less than 30 min while in healthy subjects some allergen persisted up to 48 hours. [26] [27]. Consistent with these findings transport of intact albumin (125-I-HSA) across nasal epithelium was shown in minutes in 9/10 patients with allergic rhinitis vs. 3/9 control subjects also suggesting disease related differences in the elimination of the allergen [28].

The purpose of the current study was to further characterise the interaction between allergen and the nasal cavity. The aims of this study were twofold: (1) to describe the dispersal of Der p 1 allergen in the nasal cavity of healthy nonatopic subjects by measuring the recovery of allergen following nasal administration, using a range of retrieval techniques, and (2) to evaluate whether Der p 1 can be detected in the nasal mucosa of Der p 1 sensitized or non-sensitized subjects, after natural exposure and in vivo nasal challenge as assessed by nasal biopsies.

## Material and Methods

### 1. Recovery of inhaled allergens from the nonatopic nasal mucosa

#### Study subjects

Twenty normal volunteers, aged 21 to 45 years (13 female, 7 male), participated in the study. All were nonatopic, based on skin prick testing with common inhalant allergens, and had no history of rhinitis. Nonatopic subjects instead of atopic subjects were selected since atopic subjects would experience an immediate allergic response and expel the Der p 1 allergen by sneezing and rhinorrhoea thus making sample collection inaccurate.

#### Ethics statement

Before the study commenced, approval was obtained from the Human Ethics Review Committee of the University of Sydney (protocol number 01/02/17) and written informed consent was obtained from each subject.

#### Experimental design

All subjects had **allergen challenges** performed on three separate days, between 8 and 15 days apart. The allergen was administered to the nasal cavity as an aqueous solution on two occasions (Nasal Aqueous challenge) and as household dust particles on one occasion (Nasal Dust challenge). To examine the time course of allergen recovery after administration to the nose, sampling was performed at different time points after the challenge.

**Nasal and sputum samples** were always collected in the same sequence of nasal wash followed by 5 minutes of nasal mucus collection and then by 10 min of sputum induction. This sampling sequence was commenced at 1, 5, 10 or 15 minutes after the allergen challenge, with different commencement times used in different subjects.

**A gargle**, to sample the oropharyngeal compartment, was performed at times varying from 30s to 30min after challenge

To determine the retention of **non-allergenic particles** in the nose, six subjects had 6 mg of 40μm diameter carbon particles (approximately 3 x 10^9^ particles; Sigma-Aldrich Pty Ltd, NSW 1765, Australia) inserted into one nostril using a nasal powder applicator (Teijin Ltd). Samples were collected by nasal wash, gargle, mucus collection and sputum induction, starting one minute after the insertion of the particles.

#### Nasal allergen challenge

Subjects inhaled maximally; then the aqueous allergen or household dust was inserted into one nasal cavity during a 10 second breath hold. Subjects then breathed orally for five breaths with their head bent forward and their nose occluded.

**Nasal aqueous challenges** were performed by spraying 0.2ml of an aqueous solution of mite extract (Dermatophagoides pteronyssinus 10,000AU/mL, Bayer Hollister-Stier, Richard Thompson, Sydney) one 0.2 ml dose containing a mean of 2347ng (95% CI 2077–2617) of Der p 1 allergen, into one nostril only, using a nasal pump spray bottle (supplied by Schering Plough). Reproducibility of the nasal aqueous challenges was tested by repeated challenges on 39 occasions and the 95% limits of agreement for the percent total recovery ranged from 12 to 16%.

**Nasal dust challenges** were performed using fine dry household dust administered by a nasal powder applicator (Teijin Ltd). A small clear capsule (Capsugel, Australia) containing 6 mg of house dust with a mean of 2329 ng (95%CI 1938.27 to 2721.33) of Der p 1 allergen was placed inside the applicator and pierced. The applicator outlet was placed in front of the selected nostril, and puffed into the nostril. Negligible dust remained in the capsule after the challenge.

#### Sample collection

One to fifteen minutes after the challenge ***nasal wash*** was performed; the nasal cavity was sampled by lavage using 15 ml of normal (0.9%) saline. The liquid was administered into the nose by holding a nasal pool device [29] firmly to the nostril, squeezing so the contents entered the nasal cavity, holding this position for 1 minute and then allowing the bottle to re-expand which retrieved the liquid back into the bottle. Patients were orientated to tilt their head forward and towards the side to avoid fluid spilling into the nasopharynx. The retrieved volume was measured and samples were then stored at—20°C. Most of the 15ml volume inserted for the nasal wash was recovered. The median volume of lavage fluid recovered from the nasal washing was 11.5 ml (IQR = 2.5).

**To collect the nasal mucus** the subjects were asked to blow their nose repeatedly into a specimen collection jar for 5 minutes following the nasal wash [6]. Median volumes of nasal mucus were 4 ml (IQR = 2).

Normal saline (5ml) was added to the collection jar and mixed with the mucus for 30s using a vortex mixer. Liquid phase was then separated from the mucus plugs, which were liquefied by the addition of equal amount of Sputolysin (dithiothreitol; 10% Sputolysin, Calbiochem Boehring; diluted with distilled water to final concentration of 1%) and rotated at room temperature for 30 minutes and then centrifuged at 4°C at 407 x g for 10minutes [30]. The liquid phase and the supernatant were combined and then stored at—20°C. **Gargle**: To sample the oropharynx, subjects gargled 10ml normal saline for 30s, after which the resulting combination of saline and saliva was returned to a beaker, mixed for 30s with a vortex, and samples stored at—20°C.

For collecting the **sputum** a modified sputum induction [31] adapted for normal people was performed by using 4.5% sodium chloride solution ultrasonically nebulised (Ultra-sonic De Vilbiss 2000 nebulizer) for 10 min. Median volumes of induced sputum were 3 ml (IQR = 2.1).

The induced sputum was mixed with equal volume of saline containing 1% Sputolysin, rotated at room temperature and centrifuged for 10 min, at 4°C, 407 x g and the supernatant was stored at—20°C.

#### Measurement of allergen

The concentration of mite allergen Der p 1, in both the mite extract and house dust used for challenge, was measured using a commercial ELISA (Indoor Biotechnologies, Der p 1 ELISA kit, EL-Dp1; sensitivity 0.8 ng/ml) as previously described [32]. The concentration of allergen administered was measured prior to each challenge. **The amount of allergen recovered** per compartment (nasal wash, nasal mucus, gargle, and induced sputum) was expressed as a percentage of the total inserted allergen for each compartment, and was referred to as % recovery per compartment. The sum of these was expressed as the % total recovery.

#### Factors affecting recovery of Der p 1

The effect of a variety of diluents (saline, saline/BSA, PBS, PBS/BSA 1%) on Der p 1 recovery was studied. Percent recoveries of Der p 1 were, respectively: 83, 70, 49 and 62. Physiological **normal saline was chosen to be the diluent** of the samples since it was used for nasal challenge and collection of all other samples.

To determine if the Der p1 assays were affected by exposure of the allergens to **biological fluids**, mite extract containing 6000ng of Der p 1 allergen was added to 10ml saline (n = 5), 5ml nasal mucus (n = 5) and 5ml sputum (n = 3), incubated at room temperature for 30min and then assayed by ELISA, and the percent recovery calculated; Respectively the recoveries were: 81% (IQR = 3.22), 87% (IQR = 7.82) and 72% (IQR = 5.0); (p = N.S.).

The effect of Sputolysin treatment on the measurement of Der p 1 was studied using different concentrations of Sputolysin. A recovery of 89% was obtained using a concentration of 10% Sputolysin. With comparison to allergen recovery from normal saline (81%) these results suggest that Sputolysin per se has a significant effect on the ELISA results, but gives a better recovery than using saline only as a diluent. A final concentration of 1% Sputolysin was used throughout the study.

#### Counting of carbon particles

Carbon particles in the samples were counted using a haemocytometer.

### 2. Interaction of Der p 1 with cells in the nasal mucosa

#### Study subjects

Eight subjects (5 female, 3 male) between 13 and 55 year-old without current respiratory disease or other major illness, undergoing elective nasal surgery, were recruited for the study. Subjects with a diagnosis of asthma or smokers were excluded. Skin prick testing to a panel of 14 inhalant allergens (Bayer Hollister-Stier; Richard Thompson, Sydney) was performed in order to define their atopic status. A positive SPT was defined as a wheal diameter >3mm read at 15 minutes. Three were nonatopic and 5 atopic of whom 4 were sensitized to Der p 1 and 1 to cockroach only. All 5 atopics had clinical rhinitis and three also had nasal mucosal polypoidal changes. Antihistamine and steroid medication was withheld for 4 weeks before the biopsy was taken.

#### Ethics statement

Before the study commenced, approval was obtained from the Human Ethics Review Committee of the University of Sydney (protocol number 01/02/17) and written informed consent was obtained from each subject.

#### Biopsies

A single biopsy sample from the inferior or middle turbinate of each nostril was obtained, using Blakely forceps, after anaesthesia with cophenylcaine forte (Paed Pharm, WA). Both the region from where the biopsy was taken and the nostril to be challenged were randomly chosen. Each patient was their own negative control; a biopsy was taken from one nostril, which was challenged and a control biopsy from the contralateral nostril not challenged.

#### Preoperative nasal allergen challenge

Thirty to 60 min before the surgery, 0.2ml of an aqueous solution of mite extract (Standardized aqueous Dermatophagoides pteronyssinus allergen extract; neat extract 10,000AU/mL = 11735 ng/mL Der p 1 allergen; Bayer Hollister-Stier, Richard Thompson Sydney) was delivered via a pump spray into one nostril. The concentration of Der p 1 in the spray corresponded to 1/10 of the concentration of Der p 1 which produced a skin wheal size of 3mm in each of the atopic patients. The concentrations used in atopic subjects varied between 10–1000 AU/ml (11.74–1173.5 ng/mL). In nonatopic patients the concentration of Der p 1 used was 10,000 AU/ml (neat extract; standard protocol; 0.2 ml contained mean of 2347ng (95% CI 2077–2617) of Der p 1 allergen). The biopsy specimens were stored in PBS at 37.5°C for 90 min, placed in labelled cassettes, fixed in absolute alcohol for three hours, followed by 15 min in 95% alcohol, and 10 min in 70% alcohol. The samples were then embedded in paraffin.

#### Immunostaining

the integrity of the nasal epithelium was examined by staining one slide of each block with haematoxylin and eosin. Paraffin blocks containing the nasal tissue were cut in sections of 6μm each and mounted on gelatinised slides. For the detection of either (a) Der p 1 and macrophages (CD68) or (b) Der p 1 and IgE on the same slide, the double staining method described by Allen [33] was used. Briefly, after deparaffinizing the sections in xylene and hydrating in graded alcohols, endogenous peroxidase activity was blocked with a 6% solution of hydrogen peroxide in methanol. Using the pressure cooker method, antigen retrieval was performed using citric acid/ EDTA solution in pH 8.0. Tris-buffer (TBS) pH 7.5 was used as the washing solution.

The presence of Der p 1 in the tissue was shown by incubating the slides with Anti-Der p 1 (mouse Ab, MA-5H8, 2mg/ml, Indoor Biotechnologies) diluted 1:400 as the first primary antibody. As a blocking agent non-allergic human serum (gift from Allergen group of Woolcock Institute of Medical Research, Sydney, NSW Australia) was added to the anti-Der p 1 in the concentration of 2%. The detection system used was a universal HRP polymer kit (Advance HRP, Code K4069, DAKO Cytomation, gift from L. Yong, Sydney, NSW) followed by 4 min incubation with diaminobenzidine (DAB) as a chromogen. After the staining the slides were left in TBS over night. On the second day the slides were microwaved for 10 min, and rinsed in TBS. Then a second primary antibody, either (a) anti-CD68, diluted 1:100 (monoclonal mouse anti-human CD68, clone KP1, code M0814, DAKO Cytomation; biotinylated using Dako ARK, code K3954), for the detection of macrophages, or (b) anti-IgE, diluted 1:2000 (polyclonal rabbit anti-human IgE, code A0094; gift from S. Davis, Sydney, NSW, Australia), was used. The detection systems for anti-CD68 and anti-IgE, were respectively, alkaline phosphatase kit (VECTASTAIN ABC-AP Universal, code AK-5200) and goat anti-rabbit peroxidase polymer (DAKO EnVision, Peroxidase, Rabbit, code K4003). The chromogens were, respectively, liquid permanent red (DAKO Cytomation, code K3467 and 3-amino-9-ethylcarbazole (AEC+, DAKO Cytomation, code K3461). The counterstaining was made using haematoxylin.

**The specificity of the immunohistochemical stainings** was controlled using both an irrelevant isotype and omitting the antibodies. Nonspecific binding of antibodies to nasal mucus could be excluded by negative staining for IgE in biopsies from nonatopic subjects.

#### Immunohistochemical evaluation

using light microscopy, the distribution and localization of cells and tissues positive to antibodies to Der p 1, CD68 and IgE were defined, qualitatively. The cells stained positive were counted by two independent readers. Photomicrographs of five fields of each section, in three different days of staining, were taken and Der p 1 staining cells were counted with reproducible results (CV less than 20%).

**Haematoxylin and eosin staining** was performed on each tissue section and the number of eosinophils was counted.

#### Data analysis

The amount of allergen recovered was expressed as a percentage of the total allergen inserted. These values were non-normally distributed, so non-parametric tests were used for the data analysis and the results are expressed as median and interquartile range (IQR). Comparisons of allergen recovery between types of challenge, between time points and between sampling sites were made using analysis of variance (Friedman test for multiple samples), Wilcoxon rank sum test or Wilcoxon signed rank test. A p value < 0.05 was regarded as significant. Reproducibility of the ELISA measurements was tested by coefficient of variation (CV%). Values less than 20% were regarded as acceptable.

To evaluate the significance of the changes in cell numbers after the allergen challenge, Der p 1 or CD68 staining the cells and eosinophils were counted and the differences between challenged and control samples were analysed using Student’s t-test. A p value < 0.05 was regarded as significant. An ordinal scale was used to define intensity of Der p 1 staining in nasal tissue biopsies obtained before and after allergen challenge. The ordinal type allows for rank order (negative, 1+, 2+, 3+, 4+), but does not allow for measuring relative degree of difference between them.

## Results

### 1. Recovery of inhaled allergen

Preliminary challenge studies indicated low recovery of Der p 1 from the nasal samples. When studying the factors affecting the recovery of allergen in the ELISA system, saline/0,1% Tween proved to be the best diluent (102% recovery), but saline was chosen since it felt to be the safest to be used for the *in vivo* challenges. The allergen in saline stored in glass or plastic tubes for a week (+4°C) retained 90% of Der p 1 indicating some binding to the test tubes. There was no significant reduction in Der p 1 levels when samples were stored at +4°C in saline for less than 2 weeks.

When investigating the effects of nasal mucus or induced sputum on the recovery of Der p 1, losses of 13% and 28%, respectively, were observed. Dithiothreitol (Sputolysin in saline, final concentration 1%) used to liquefy mucus (27) as such did not affect Der p 1 recovery.

Carbon particles were used as an inert control for particulate dust challenge. The median percent recoveries of carbon particles in the gargle, nasal wash, nasal mucus and sputum were medians 0.1% (IQR = 0.2), 3.9% (IQR = 3.2), 5.5% (IQR = 3.3) and 0.1% (IQR = 2.1) respectively. The median of total percent recovered was 9.26% (IQR = 2.7), (data not shown).

After allergen challenge, the study subjects were randomized to different time points for sample collection. The total percent of Der p 1 allergen recovered was consistently low and similar throughout the different time points for collection. Table 1 presents the number of subjects per time point and the % total amount of allergen recovered at each time point. Although allergen was already recoverable at 1 min, there were no significant differences in the total allergen recovery at 1, 5, 10, or 15 minutes (p = 0.2; analysis of variance, Friedman test for multiple samples) after administration of allergen. Furthermore, there were no significant differences in allergen recovery at any time point whether the Der p mite allergen challenge used was aqueous or particulate (p≥0.2; Wilcoxon rank sum test).

**Table 1:** Total recovery of Der p 1 after allergen challenge in 20 nonatopic subjects.

Type of challenge****	Time sampling commenced after challenge***			
	1 min	5 min	10 min	15 min
**Nasal aqueous** (N)*	13	9	10	7
Total recovery % (Median)	8.4	17.7	8.0	7.8
(IQR)	8.0	8.0	5.0	4.0
**Nasal dust** (N)**	7	5	5	5
Total recovery % (Median)	6.2	11.8	8.5	4.5
(IQR)	6.6	1.6	13.4	4.8

*Each subject had two random sampling times

**Samples taken on one occasion only.

***Total allergen recovery—comparison between sampling times (p = 0.2; analysis of variance, Friedman test for multiple samples)

**** Total allergen recovery—comparison between aqueous or particulate at any time point (p≥0.2; Wilcoxon rank sum test)

N = Number of subjects; IQR = Interquartile Range

The total amount of allergen recovered from nasal wash, nasal mucus, gargle and sputum samples expressed as a percentage of the amount administered, was less than 25% under any of the conditions. The effect on allergen recovery of the time course of allergen passage through the nasal cavity was investigated by starting collection at different times after allergen administration, however this made no difference to the amount of allergen recovered.

**Fig 1:**
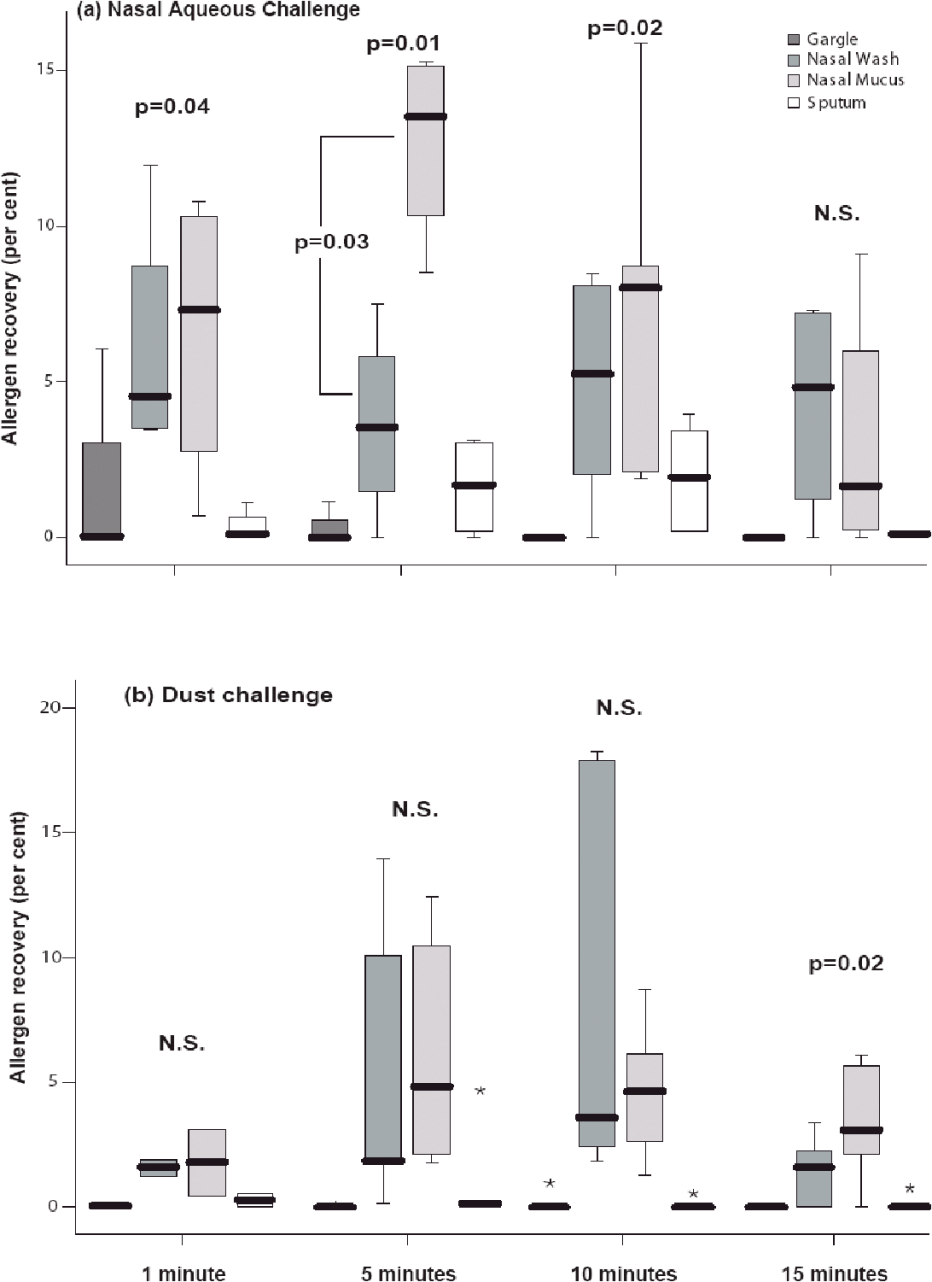
Percent recovery of allergen from different compartments.

The mite allergen recovered from each compartment: nasal wash, nasal mucus, gargle and sputum samples after an aqueous or particulate mite allergen nasal challenge, is presented in Fig 1. The amount of allergen recovered from the nose at times 1, 5 and 10 minutes after nasal aqueous challenge was highest in the nasal mucus compared with the nasal wash or lower respiratory tract samples (gargle, induced sputum; p = 0.04, p = 0.01, p = 0.02, respectively; Friedman test for multiple samples). A significantly higher amount of allergen was detected in nasal mucus compared to nasal wash at 5 min after aqueous challenge (Fig 1A; p = 0.03; Wilcoxon signed rank test). When all the different compartments and sampling times were compared the highest percent of allergen recovery (median = 12.0%, IQR = 4.4) was into the nasal mucus compartment five minutes after the nasal aqueous allergen challenge (p = 0.01) (Fig 1A). When combined allergen recovery from the upper respiratory tract (nasal wash and nasal mucus) was compared with sampling from the oropharynx and lower respiratory tract (gargle and induced sputum) much greater amounts of allergen were obtained from the upper airway or nose for both aqueous (p<0.001) and particulate (p<0.001) challenges (analysis of variance, Friedman test for multiple samples).

Recovery of Der p 1 after (Fig 1A) nasal aqueous and (Fig 1B) nasal dust challenges (median and IQR = Interquartile Range). Statistical differences between the different sampling methods (gargle, nasal wash, nasal mucus, sputum) were determined using analysis of variance (Friedman test for multiple samples). The comparison between nasal mucus and nasal wash (Fig 1A, 5 min) was analyzed using Wilcoxon signed rank test. The asterix (*) denotes individual outliers.

### 2. Interaction of Der p 1 with the nasal mucosa

**Fig 2:**
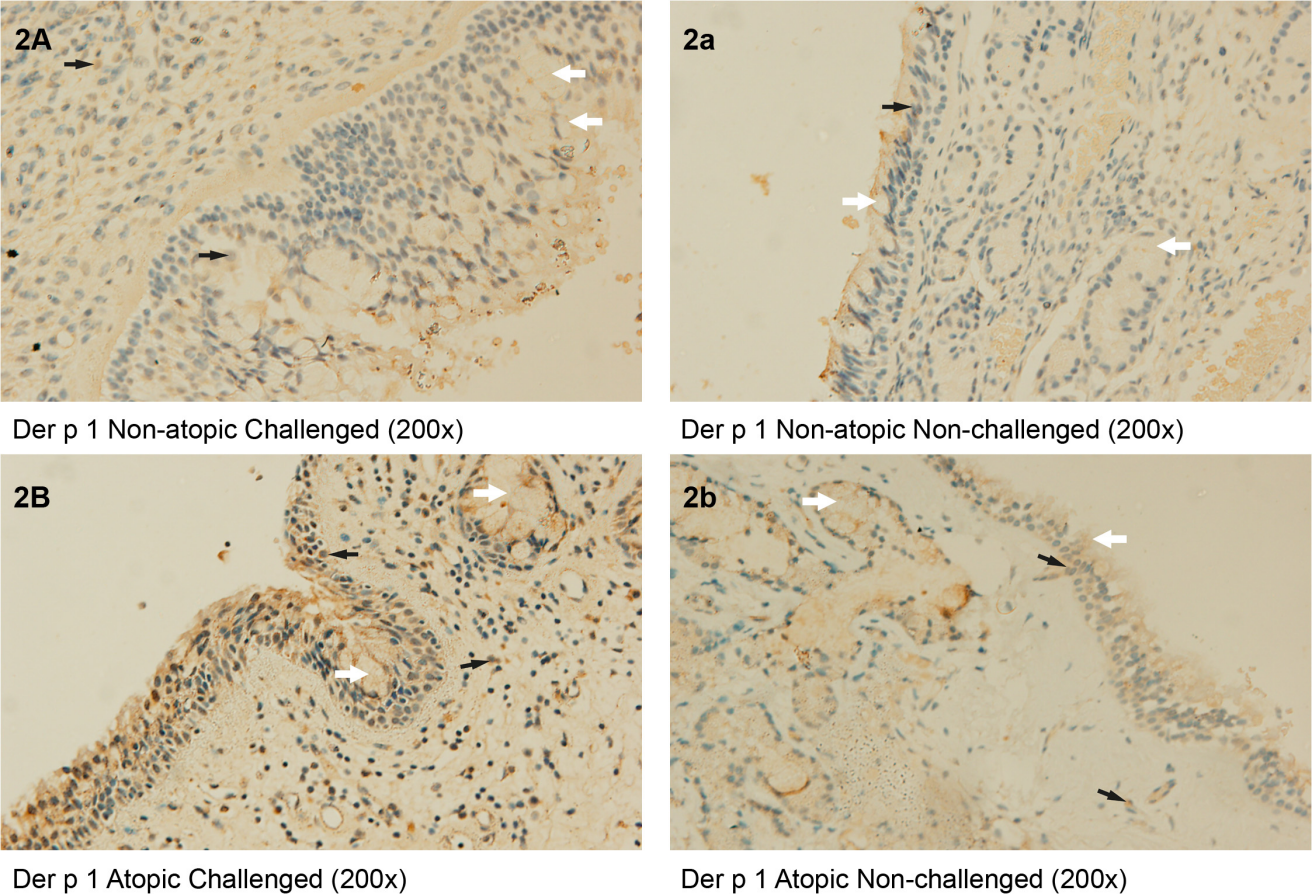
Immunohistochemical staining of Der p 1 using mouse anti-Der p 1.

The immunohistochemical staining of Der p 1 was found in all the samples obtained from atopic (n = 5) and nonatopic (n = 3) patients (Table 2). In the unchallenged samples there were minimal differences between atopics and nonatopics in the weak positive Der p 1 staining of the nasal mucosal epithelium or lamina propria (LP), while stronger staining was observed only in the subepithelial mucous glands of atopics. There were no significant differences between atopics and nonatopics in the baseline number of Der p 1 stained cells in any of the tissue compartments. (Table 2; Fig 2A and 2B).

**Table 2:** Immunohistochemical detection of Der p 1, IgE and CD68, and the presence of eosinophil leukocytes in nasal biopsies before and after *in vivo* challenge with Der p 1 allergen.

Immunohistochemical staining		Atopic (n = 5)			Nonatopic (n = 3)			
		pre	Post	p***	Pre	Post	p***	p****
Der p 1 staining in tissue**	EP	2+	3+	NA	2+	3+	NA	NA
	GL	3+	4+	NA	2+	3+	NA	NA
	LP	1+	3+	NA	1+	2+	NA	NA
Der p 1 positive cells/mm2	EP	20 (2.5–27.5)	75 (45–115)	0.11	32 (25–45)	65 (50–100)	0.32	0.27
	LP	19 (2.5–30)	117 (40–220)	0.24	32 (22.5–47.5)	55 (45–72.5)	0.32	0.43
CD68 positive cells/mm2*	EP	61 (40–72.5)	88 (22.5–290)	0.45	41 (25–52.5)	57.5 (55–60)	0.4	0.45
	LP	103 (80–198)	146 (95–425)	0.52	169 (153–183)	118 (103–128)	0.4	0.43
IgE positive cells/mm2*	EP	43 (25–72.5)	40 (15–60)	0.97	0	0	NA	0.22
	LP	82 (37.5–123)	30 (22–35)	0.16	0	0	NA	0.08
Eosinophils/mm2	EP	1 (0.75–1.0)	6 (0.5–15.5)	0.39	0	0	NA	0.01
	LP	11 (4.5–17.3)	95 (12.5–258)	0,41	4 (2.25–4.75)	5 (4.25–5.5)	0.46	0.16

H-E staining for Eosinophils; see Methods.

*Double staining with Der p 1.

** Staining of cells and the mucous in the epithelium, lamina propria and the submucosal glands. An ordinal scale was used for intensity of Der p 1 staining. This allows for rank order (negative, 1+, 2+, 3+, 4+), but does not allow for measuring relative degree of difference between them.

***Comparison between non-challenged (pre) and challenged (post) tissue.

****Comparison between atopic and nonatopic (non-challenged tissue).

EP: nasal mucosal epithelium; LP: lamina propria; GL: subepithelial mucous glands.

Number of cells: Mean (range); NA: not available; Statistics: t-test was used for comparison between means.

Samples of nasal mucosa from healthy (2A, 2a) and allergic (2B, 2b) subjects, from challenged (2A, 2B) and the non-challenged contralateral nostrils (2a, 2b). (200x). Note identical staining of Der p 1 in the mucous of the epithelium and in the subepithelial mucous glands of non-challenged tissues of nonatopics (2a) and atopics (2b), but stronger staining after challenge of both the mucous compartment, the epithelium and the lamina propria in atopic nasal mucosa (2B). (White arrows = Der p 1 in the mucous of the epithelium and subepithelial mucous glands; black arrows = Der p 1 in the epithelial cells and lamina propria).

Following nasal challenge Der p 1 staining was increased in all tissue sections from atopic subjects. There were increased numbers of Der p 1 positive cells in the epithelium (from 20 to 75/mm^2^) and in the lamina propria (from 19 to 117/mm^2^). Some of this staining was co-localized with IgE-positive cells, eosinophils and macrophages (Table 2; Fig 2B). Also in nonatopics, after nasal allergen challenge, an increase in the Der p 1 staining at all tissue compartments was observed, albeit not to such a great extent—despite use of higher allergen concentration than in atopics. (Table 2; Fig 2A).

CD68 positive cells of the monocyte/macrophage lineage were numerous in the non-challenged tissue from both the atopic (EP 61/mm^2^; LP 103/mm^2^) and the nonatopic (EP 41/mm^2^; LP 169/mm^2^) subjects. An increase in the number of CD68 positive cells was observed in the nasal mucosal epithelium and the lamina propria of the atopic (EP 88/mm^2^; LP 146/mm^2^) and in the epithelium of the nonatopic (EP 58/mm^2^; LP 118/mm^2^) subjects after nasal challenge (Table 2).

In the challenged tissue from atopic subjects, IgE positive cells were observed in subepithelial lamina propria, and found also amongst epithelial cells at the mucosal surface (EP 40/mm^2^; LP 30/mm^2^); (Table 2). The control tissue, from the contralateral nostril, showed the same degree of positivity to IgE in the epithelium, but due to wide variability the mean number of IgE positive cells was higher than in the challenged tissue deeper in lamina propria (EP 43/mm^2^; LP 82 (37–123)/mm^2^). As expected, in nonatopics, IgE positive cells were absent both in the challenged and control tissue.

Haematoxylin and eosin staining of the unchallenged tissue of atopic subjects allergic to Der p 1 showed low number of eosinophils (EP 1/mm^2^; LP 11/mm^2^), which 30 minutes after the nasal allergen challenge increased almost 10-fold (EP 6/mm^2^; LP 95/mm^2^) (Table 2). Eosinophils were more common in Der p 1 positive/ intense areas indicating a response to Der p 1 challenge. As expected significantly more eosinophils were found in the epithelium of samples from atopic subjects, since no eosinophils were observed in the epithelium and only a few in the lamina propria from nonatopics (before 4/mm^2^ and after challenge 5/mm^2^, respectively).

## Discussion

Recently there has been an increased interest in the way in which allergens interact with the epithelium and the differences that exist between different allergens, in addition to epithelial related differences between atopics, nonatopics, asthmatics and nonasthmatics [34]. Our data extends these observations by showing that Der p 1 allergen either soluble or particulate, administered intranasally to healthy nonatopic individuals, is retained in the nasal cavity such that less than 25% of the total allergen inserted could be retrieved after challenge. Although this finding may seem self-obvious, as far as we know, this is the first study to insert and then attempt to quantify the recovery of allergen from the upper respiratory tract. Our findings were substantiated by positive immunohistochemical staining of Der p 1 in the nasal mucosal epithelium, subepithelial mucous glands and lamina propria after natural exposure and increased staining after nasal challenge independent of atopic status.

It is not known for how long allergen may persist in the nasal cavity. Also the precise way in which the nose handles intranasal allergen is unknown. Our results in healthy subjects show that the allergen is retained in nose within minutes irrespective of whether an aqueous or particulate challenge is used. Rapid elution, within 1 min of Der p 1 allergen from mite faeces was observed by Tovey et al. [35], which may explain why no difference between the aqueous and particulate allergen challenges was found. When different compartments and sampling times were analysed the highest recovery was in the nasal mucus. Our results are in keeping with those of Passalacqua et al. [26] who showed 30 min (up to 24hr) persistence of radiolabelled Par j 1 allergen in the nasal mucosa of healthy subjects. Similarly, Bagnasco et al. [27] showed that up to 10% of the allergen inserted persists in the nasal cavity of healthy subjects for up to 48h. Thus nasal mucus/mucous membrane may serve as a reservoir enabling subsequent allergen interaction and transport through the respiratory epithelium or later elimination by mucociliary clearance and gastrointestinal processing.

Our finding of low recovery of the inserted Der p 1 may have been confounded by the recovery methods at different stages of the process. The nasal pool device and other lavage processes such as bronchoalveolar lavage techniques are standard research protocols and fluid loss using these methods is expected [29, 31]. Using saline diluent as a control would have allowed volume loss during the procedure to be determined. Inert carbon particles served as a particulate control and also demonstrated low recovery rates indicating that the procedure itself was adequate. The ELISA assay used to measure Der p 1 was not intended for measurements in nasal mucus and sputum containing cellular degradation products, DNA, substances secreted by airway epithelial cells (including proteases, soluble receptors and binding proteins), which like any biological fluids may have matrix effects in assays by alteration of pH and ionic strength. Dithiothreitol (DTT) reducing disulphide bonds in polymeric mucins, allowing cells and debris to be released, was used for dispersal of the mucus [30, 36]. Theoretically DDT might interfere with disulphide bonds present in the capture antibodies used in ELISA. Therefore, spiking experiments were performed to exclude these potential confounding factors. The commercial ELISA used for measuring Der p 1 [32] proved accurate and repeatable. However, our *in vitro* experiments showed that some allergen was lost during the procedure as shown by spiking experiments and also use of DTT resulted in 10 per cent allergen loss, which is in the limits of acceptable recovery [36]. All samples were assayed within 2 weeks of collection which minimised storage related loss of Der p 1. Taken together, these methodological and technical issues would account for at most 20–30% loss of the allergen, but not enough to discount our results.

Our study of allergen retrieval after local application of high doses of allergen remains an imperfect approximation of real-life constant exposure to low dose of allergen, which happens in day-to-day life. Therefore, the results need to be interpreted with caution. However all nasal allergen challenges performed in atopic rhinitis studies use similarly high concentrations to produce symptoms. It would be interesting to study atopic rhinitic subjects but it would be difficult to account for allergen loss due to symptoms of anterior rhinorrhea and sneezing following intranasal insertion of allergen. Also the numbers of atopic and nonatopic subjects participating in the biopsy study were too small to allow definite comparison between groups, especially when three out of five atopic subjects had polypoidal mucosal changes associated with perennial rhinitis, often coinciding with eosinophil infiltration [1, 19].

Overall less than 25% of allergen inserted intranasally was retrievable using nasal lavage, nasal mucus collection, gargle and sputum induction. Less Der p 1 allergen was quantified in gargle and induced sputum compared to nasal lavage and nasal mucus. This could be a function of the loss of sample associated with the retrieval methods for these respective compartments. It is likely that allergen could be handled by the mucociliary escalator [37], and it is possible that a gargle was unable to sample this compartment effectively. Similar results were obtained with inert carbon particles suggesting that binding to nasal mucus—where most of the allergen was located—may be a non-specific mechanism. Hence over 75% of inserted allergen remains inside the nose using our model. This is likely to be an overestimate as others have demonstrated that there is clearance of allergen via the mucocliliary escalator into the gastrointestinal tract and uptake by the vascular system. We did not undertake studies with labelled allergen but others have done so. A mouse model, which eliminated gastrointestinal processing by esophageal ligation, showed that biotin-labelled ovalbumin administered either intranasally or intrabronchially demonstrated both tissue and plasma associated allergen at 15 minutes post exposure [38]. Also human studies using Par j 1 allergen and radiolabelled human serum albumin showed rapid passage, under 30 minutes, through the epithelium as intact allergen was detected in the blood, and there was also a later peak occurring due to gastrointestinal absorption [26, 28]. We did not continue sample collection for a longer period than 15 minutes but our data showed that there was already reduced yield at that time compared with earlier sampling indicating rapid trapping of allergen within the nasal mucosa.

Exposure to allergens and irritants increases mucus production by goblet cells in the epithelium and by the subepithelial mucous gland cells [9, 39], and increased mucus production has been obeserved in athmatic subjects [40]. There are no studies of allergens being trapped by the secreted mucin raft (7–70 μm depth; containing MUC5AC from goblet cells and MUC5B from mucous glands) or by the cell surface bound tethered mucins (MUC1, MUC4, MUC16), but our immunohistochemical data in atopic and healthy subjects suggest that Der p 1 is bound to nasal epithelial cells and mucus. Since mucus can bind many substances we needed to show our stainings were specific; non-specific binding of antibodies by the mucus was excluded by using appropriate controls: omitting the specific antibody, using monoclonal antibodies of irrelevant isotype or using the secondary antibody of irrelevant specificity.

The current study expands our knowledge of the in vivo data on the interaction between allergen and the nasal mucosa and shows that allergen is retained in the nasal cavity mainly associated with mucus and that Der p 1 allergen is detected in both the epithelium and lamina propria. Previous in vivo studies have shown during challenge with grass pollen allergens, a rapid (30 min) increase in antigen presenting CD1a^+^ Langerhans cells in the nasal epithelium, observed in allergic subjects but not in healthy controls [41]. Similarly there was an increase in the activated CD68^–^, CD123^+^ plasmacytoid dendritic cells (DC) in the nasal sub-epithelium 24 hours after birch or timothy pollen allergen challenge in subjects with allergic rhinitis [42]. These data support the notion that allergens reaching the epithelium rapidly cross the epithelial barrier and bind to IgE-positive cells (mast cells, DCs, eosinophils, basophils and monocytes) [43] and activate the innate immune system [4, 44].

Our demonstration, in atopic and less markedly in nonatopic subjects, of strong Der p 1 staining in the mucosal epithelium, subepithelial mucous glands and lamina propria 30 to 60 min after nasal allergen challenge would support the existence of specific mechanisms for transport of Der p 1 through the nasal epithelium to the subepithelial site in allergic subjects. Indeed Grieff et al. [45] demonstrated no increased nasal permeability to a small peptide marker in symptomatic HDM allergic rhinitics supporting the concept that entry of allergen into the subepithelial space does not depend on an underlying increase in epithelial permeability in atopics but more likely on specific receptor and transport mechanisms. While our study was not designed to examine this possibility there is data supporting this concept. Allergens of house dust mite include several with proteolytic activity including cysteine proteases [46, 47] and Der p 1 has been shown to increase permeability of the epithelium in vitro by degrading tight junction proteins occluding, claudin, and desmoplakin [8, 48, 49, 50]. Thus these activities may facilitate access of Der p 1 allergens through the nasal epithelium using altered intercellular tight junctions as the gateway.

Although protease activity of Der p 1 may explain increased paracellular penetration of Der p 1 through the respiratory epithelium, it does not explain our findings suggesting higher penetration in atopic than nonatopic subjects. In addition to affecting intercellular tight junctions, the cysteine protease allergen, Der p 1, stimulates human respiratory epithelial cells directly by activating the protease–activated receptor 2 (PAR-2) [51] resulting in release of proinflammatory cytokines (IL-6 and IL-8) and prostanoids (PGE2) [52] as well as disrupting E-cadherin mediated cell-cell contacts. PAR-2 can also be activated by other allergens such as cockroach, *alternaria*, *aspergillus* and pollen. [14, 17]. Interestingly, PAR-2 is upregulated in patients with asthma [53] and in the nasal mucosa of subjects with seasonal allergic rhinitis [54, 55]. Similarly, in response to HDM allergens gene expression profiles of epithelial cells cultured from nasal mucosal biopsies of allergic subjects are upregulated compared with healthy subjects favouring an inflammatory response [14]. Both healthy and allergic subjects are exposed to HDM constantly, therefore, these results suggest that the nasal epithelium of allergic subjects stays in an activated state because of an inability to shut this response down [2, 3]. These findings indicate that PAR-2 mediated changes could be an important pathway to increase allergen transport in atopics.

Likewise non-proteolytic cat and dog allergens directly activate respiratory epithelial cells by protease-independent mechanisms [56]. In addition, direct uptake and transcytosis of Phl p 1 by airway epithelial cells in less than 2 hours suggests that alternative mechanisms exist [25]. Indeed, a specific epithelium cell uptake and transport system for birch pollen allergen Bet v 1 has been shown [57], and it is not known whether this may apply to house dust mite allergens also.

Airway epithelial cells are no longer considered to act only as a physical barrier towards inhaled allergen, but also to actively contribute to airway inflammation by detecting and responding to environmental factors [2, 10, 11]. Allergen binding to Toll like receptors (TLR) and other pattern recognition receptors (PRR), e.g. TLR2, TLR4 expressed by respiratory epithelial cells and dendritic cells (DC) activates innate immunity or inflammatory signalling pathways thus facilitating the interaction of Der p 1 with the nasal mucosa [11, 12, 13, 58, 59]. Since many allergens are glycoproteins carbohydrate recognizing PRRs expressed on airway epithelial cells are likely to be involved (2). A recent study shows that glycosylated major allergens like Der p 1, Fel d 1 (*Felix domesticus*), Ara h 1 (*Arachis hypogaea*), Der p 2 (*Dermatophagoides pteronyssinus* group 2), Bla g 2 (*Blattella germanica*) and Can f 1 (*Canis familiaris*) all bind more efficiently than their deglycosylated counterparts to mannose receptors (MR) on DCs [60]. Less thymic stromal lymphopoietin (TSLP), an IL-17 like cytokine, which drives DC maturation for Th2 immune responses, [61], was secreted by epithelial cells exposed to deglycosylated allergens suggesting that also epithelial cells harbour MRs [60]. Dominant sugars in the glycosylated allergens are 1–2, 1–3, 1–6 mannoses. Thus the degree of mannosylation is one of the factors determining whether the protein is an allergen like Der p 1, Papain and Bromelain, while their structurally similar non-allergenic counterparts Cysteine protease B, Calpain and Staphopain B are non-glycosylated and contain significantly less mannoses [60]. Since glycosylation of proteins is important in allergen recognition and uptake on the epithelial surface it could partly explain our finding of strong binding of Der p 1 to the nasal mucosa after allergen challenge.

In spite of growing evidence that many inhaled allergens activate airway epithelial cells to secrete cytokines which drive DC maturation to promote Th2 immune responses we still do not know how well this model, derived mainly from *in vivo* murine studies or *in vitro* studies using human airway epithelial cell lines, mimics true life *in vivo* in humans or is true for all allergens [34, 62]. Epithelial cell lines hardly can simulate the pseudostratified airway epithelium with multiple cell types and overlying mucus and surface lipids. Also direct evidence supporting a role for airway leakiness in mucosal allergen sensitization in still lacking.

So far there are few *in vivo* studies regarding allergen deposition in the nasal cavity in man. The purpose of the current study was to further characterise the interaction between allergen and the nasal mucosa. Our data show that either soluble or insoluble Der p 1 allergen, administered intranasally to healthy nonatopic individuals, is rapidly retained in the nasal cavity, the nasal mucus acting as the main reservoir. Although binding to mucus may be non-specific our skin prick test (SPT) studies suggest active interaction since Der p 1 allergen incubated with nasal mucus from atopic subjects with rhinitis shows an increased SPT reactivity in the same subjects [63]. Active interaction with nasal secretion of Phl p 5, a major allergen of timothy grass, was observed also by Bufe et al. [64] who showed conversion of the allergen to various forms with molecular size between 10 and 20 kDa in the presence of nasal secretions, and also showed that SPT in allergic patients using the mixture of converted peptides caused a significantly higher allergic response compared with the parent protein. In line with this study we also have shown increased allergenic activity of the allergens of *Dermatophagoides pteronyssinus* as a result of proteolytic conversion by the nasal mucus [65]. Taken together, these findings suggest that inhaled allergens may be trapped and enzymatically converted by proteases in the nasal mucus and this may enhance their allergenicity. Since significant amounts of allergen are held inside the nasal cavity this provides a potential mechanism for sensitisation, ongoing symptoms and persistent inflammation. The fact that nonatopics also retained intranasal allergen does support the possibility of local allergic rhinitis (LAR) [66]. The biopsy results show that Der p 1 accesses subepithelial tissue within 30–60 minutes, more effectively in atopic subjects than in nonatopics. This is in line with the hypothesis of the specific binding of Der p 1 to epithelial cells of nasal mucosa and subsequent transport to the subepithelial lamina propria, where it can bind to the cells of the innate and adaptive immune system or access the blood stream resulting in both local and distal organ effects.
